# Clinical Effects of Regular Dry Sauna Bathing: A Systematic Review

**DOI:** 10.1155/2018/1857413

**Published:** 2018-04-24

**Authors:** Joy Hussain, Marc Cohen

**Affiliations:** School of Health and Biomedical Sciences, RMIT University, Melbourne, VIC, Australia

## Abstract

**Introduction:**

Many health benefits are claimed by individuals and facilities promoting sauna bathing; however the medical evidence to support these claims is not well established. This paper aims to systematically review recent research on the effects of repeated dry sauna interventions on human health.

**Methods:**

A systematic search was made of medical databases for studies reporting on the health effects of regular dry sauna bathing on humans from 2000 onwards. Risk of bias was assessed according to the Cochrane Collaboration guidelines.

**Results:**

Forty clinical studies involving a total of 3855 participants met the inclusion criteria. Only 13 studies were randomized controlled trials and most studies were small (*n* < 40). Reported outcome measures were heterogeneous with most studies reporting beneficial health effects. Only one small study (*n* = 10) reported an adverse health outcome of disrupted male spermatogenesis, demonstrated to be reversible when ceasing sauna activity.

**Conclusions:**

Regular dry sauna bathing has potential health benefits. More data of higher quality is needed on the frequency and extent of adverse side effects. Further study is also needed to determine the optimal frequency and duration of distinct types of sauna bathing for targeted health effects and the specific clinical populations who are most likely to benefit.

## 1. Introduction

Sauna bathing is a form of whole-body thermotherapy that has been used in various forms (radiant heat, sweat lodges, etc.) for thousands of years in many parts of the world for hygiene, health, social, and spiritual purposes. Modern day sauna use includes traditional Finnish-style sauna, along with Turkish-style Hammam, Russian Banya, and other cultural variations, which can be distinguished by the style of construction, source of heating, and level of humidity. Traditional Finnish saunas are the most studied to date and generally involve short exposures (5−20 minutes) at temperatures of 80°C–100°C with dry air (relative humidity of 10% to 20%) interspersed with periods of increased humidity created by the throwing of water over heated rocks [1]. In the past decade, infrared sauna cabins have become increasingly popular. These saunas use infrared emitters at different wavelengths without water or additional humidity and generally run at lower temperatures (45–60°C) than Finnish saunas with similar exposure times [2]. Both traditional Finnish and infrared sauna bathing can involve rituals of cooling-off periods and rehydration with oral fluids before, during, and/or after sauna bathing.

Sauna bathing is inexpensive and widely accessible with Finnish-style saunas more often used in family, group, and public settings and infrared saunas more commonly built and marketed for individual use. Public sauna facilities can be located within exercise facilities and the relationship between saunas and exercise, which may include synergistic hormetic responses, is an area of active research [3–8]. The use of private saunas, especially involving infrared saunas, is also increasing and saunas are used for physical therapy in massage clinics, health spas, beauty salons, and domestic homes. This trend is capitalising on the call for additional lifestyle interventions to enhance health and wellness particularly in populations that have difficulty exercising (e.g., obesity, chronic heart failure, chronic renal failure, and chronic liver disease) [9]. Facilities offering sauna bathing often claim health benefits that include detoxification, increased metabolism, weight loss, increased blood circulation, pain reduction, antiaging, skin rejuvenation, improved cardiovascular function, improved immune function, improved sleep, stress management, and relaxation. However, rigorous medical evidence to support these claims is scant and incomplete, as emphasized in a recent multidisciplinary review of sauna studies [10].

There is considerable evidence to suggest that sauna bathing can induce profound physiological effects [4, 11–17]. Intense short-term heat exposure elevates skin temperature and core body temperature and activates thermoregulatory pathways via the hypothalamus [18] and CNS (central nervous system) leading to activation of the autonomic nervous system. The activation of the sympathetic nervous system, hypothalamus-pituitary-adrenal hormonal axis, and the renin-angiotensin-aldosterone system leads to well-documented cardiovascular effects with increased heart rate, skin blood flow, cardiac output, and sweating [1, 11]. The resultant sweat evaporates from the skin surface and produces cooling that facilitates temperature homeostasis. In essence, sauna therapy capitalises on the thermoregulatory trait of homeothermy, the physiological capability of mammals and birds to maintain a relatively constant core body temperature with minimal deviation from a set point [19]. It is currently unclear whether steam saunas invoke the same degree of physiological responses as dry saunas [20], as the higher humidity results in water condensation on the skin and reduced evaporation of sweat [21].

On a cellular level, acute whole-body thermotherapy (both wet and dry forms) induces discrete metabolic changes that include production of heat shock proteins, reduction of reactive oxygenated species, reduced oxidative stress and inflammation pathway activities, increased NO (nitric oxide) bioavailability, increased insulin sensitivity, and alterations in various endothelial-dependent vasodilatation metabolic pathways [22]. It has been suggested that heat stress induces adaptive hormesis mechanisms similar to exercise, and there are reports of cellular effects induced by whole-body hyperthermia in conjunction with oncology-related interventions (i.e., chemotherapy and radiotherapy) [23]; however the mechanisms by which the physiological and cellular changes induced by sauna bathing contribute to enhanced health and/or therapeutic effects is still being explored [4, 7, 8, 24–27].

The following systematic review was undertaken to explore recent research on the clinical effects of repeated dry sauna bathing (Finnish-style, infrared, or other dry sauna forms) to document the full range of medical conditions saunas have been used for, as well as any associated health benefits and/or adverse effects observed. While a small number of reviews of sauna bathing and health have been conducted in the past [1, 2, 28–30], as far as we know, this is the first systematic review of sauna and health to include both Finnish and infrared saunas. Furthermore, this review only considers effects related to regular, multiple sessions of sauna activity rather than single sauna sessions, to better reflect the use of sauna bathing as a regular lifestyle intervention.

## 2. Methods

PRISMA guidelines for conducting systematic reviews were followed, including the use of validated tools to assess the risk of bias in randomized controlled trials [70–72].

### 2.1. Eligibility Criteria

Studies of humans undergoing repeated dry sauna bathing that reported on health measures were included in the review. Studies were included for initial review if they were published in English language from January 2000 onwards and involved research in humans undergoing repeated dry sauna sessions with at least one reported health outcome. Studies involving predominantly high-humidity (>50%) wet/steam “sauna” or immersion hydrotherapy were excluded for the potential confounding mechanisms of differential sweating rates and explicit focus of this review limited to “dry sauna” interventions. Studies of partial body heating were excluded since proposed mechanisms of action may or may not be the same as whole-body heating. Studies reporting primarily animal-based, nonhuman findings were excluded given the recognized differences in end-organ (skin) structure and responses (sweating mechanisms) between animals and humans. Studies of “sauna” as a recruitment venue for potential sexual activity, primarily regarding men who have sex with men (MSM), were excluded since these studies lacked details of sauna interventions, confounding whether wet or dry interventions, and measured health metrics focused to sexual activity but not necessarily to sauna activity.

### 2.2. Search Strategy

PubMed, Web of Science, Scopus, and Proquest were initially searched with keyword “sauna” and date restrictions of January 2000–April 2017. Search dates were chosen to focus on updated findings reflecting advancing technology in both diagnostics and physiological monitoring to build upon the foundational literature of prior nonsystematic clinical reviews of sauna activity published in the early 2000s. After further restrictions of English language and humans, records were then expanded using Google Scholar, with searches for other research by key authors, searches of citations and reference lists of original and review articles, and other “related articles”. Additional searches with expanded keywords relating to sauna including “interventional study”, “whole body hyperthermia”, and “whole body thermotherapy” were also conducted with the same initial restrictions.

### 2.3. Data Extraction

Abstracts of initially identified studies were screened by investigator JH and then the complete full-text articles of potentially eligible studies were carefully screened by both investigators JH and MC for research design, population descriptive data, timing and physical details of dry sauna intervention, outcome measures, key results, and adverse effects. Discrepancies regarding inclusion of studies or data extraction were discussed until consensus was reached.

### 2.4. Assessment for Risk of Bias

Included randomized controlled trials (RCTs) were assessed for risk of bias according to the Cochrane Collaboration's tool for assessing bias and calculated Jadad et al. scores [72]. Domains of bias assessed were selection bias (by looking for random sequence generation and allocation concealment), performance bias (by published mention of blinding of participants and personnel), detection bias (by documented attempts to blind outcome assessment), attrition bias (by evaluating for incomplete outcome data), reporting bias (by any indication of selective reporting of outcomes), and other bias (e.g., conclusions not clearly supported by reported outcomes). Risk of bias was initially assessed by investigator JH as “low”, “unclear”, or “high” and then confirmed by investigator MC. Any discrepancies were discussed until consensus was reached.

## 3. Results

### 3.1. Literature Search

**Figure 1:**
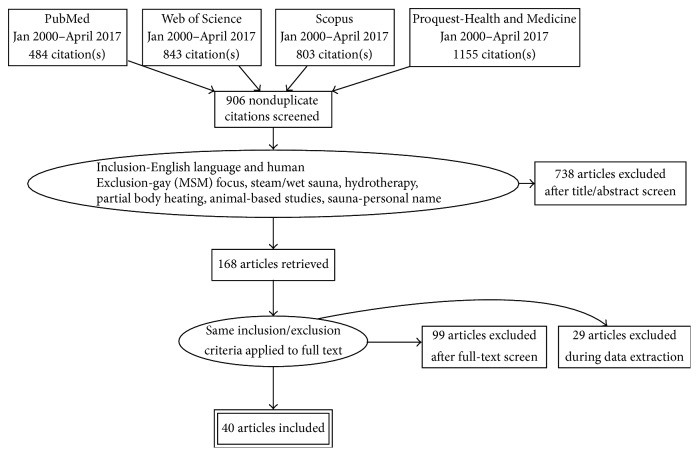
PRISMA flow diagram of evidence searches and inclusions/exclusions.

Figure 1 summarises the screening and assessment strategies used with the search results. Of the 906 nonduplicate citations initially identified, 738 were excluded after a review of the abstracts.

After retrieving 168 full-text articles and applying the same exclusion criteria as discussed above along with excluding review articles, case reports, and letters to the editor, 69 independent human studies involving dry sauna interventions were identified for further analysis.

In the data extraction step, one study was excluded since it was essentially a case series with two patients, mistakenly identified as an interventional trial conducted by a key author [73]. Another 28 studies were excluded due to the intervention being only a single session of sauna and not repeated sauna therapy, which is the stated focus of this review.

A total of 40 studies remained for inclusion in this systematic review. A summary of extracted data is presented in Tables 1–7, with tables categorised according to participant population.

### 3.2. Study Design

Of the forty studies, 13 were randomized controlled trials (RCTs), 6 were trials with nonrandomized control groups and 2 were prospective cohort studies. The remainder of studies were single-group or multigroup interventional trials (without a control group) or retrospective studies. The following three levels of evidence were used to help stratify the quality of the studies.

*Levels of Evidence*

  Level I: multicentre or single-centre, randomized controlled trial (RCT)
  Level II: controlled interventional trial; prospective cohort study
  Level III: retrospective comparative study; case-control study; pilot study.

### 3.3. Limitations/Risk of Bias

Many studies were relatively small, with limited number of participants, and a limited number of randomized studies were available for review. Of the 13 randomized controlled trials (RCTs) identified, only 3 of these studies (involving 343/840 participants) [31, 50, 58] were assessed with having a low overall risk of bias according to the Cochrane Collaboration criteria [71] and a Jadad et al. score > 3 [72]. Nine of these 13 RCTs enrolled fewer than 50 participants.  Table 8 summarises the assessments of the RCTs for overall risk of several types of bias.

The follow-up time of many of the studies was relatively short, in the order of weeks to months, thereby possibly compromising detectability and reporting of long-term health effects over years.

### 3.4. Setting and Participant Characteristics

The reviewed studies included a total of 3855 participants living in 12 different countries. Over half of the studies (22 of 40) originated in Japan. The smallest study involved Australian athletes (*n* = 7) and the two largest studies (both prospective cohort studies) involved the same cohort of 2315 Finnish men [38, 39, 61]. Most studies had small sample sizes with over half (21 of 40 studies) involving 30 or less participants.

The studies involved a range of healthy and disease populations with 6 studies of healthy individuals, 19 studies of people diagnosed with cardiovascular disease (CVD) or increased risk for CVD (i.e., congestive heart failure, type 1 or type 2 diabetes mellitus, and peripheral arterial disease), 7 studies of patients diagnosed with rheumatological, chronic pain, or mood disorders, 4 studies of patients diagnosed with airway-related disorders (i.e., chronic obstructive pulmonary disease, allergic rhinitis), 2 studies of elite athletes, and 2 studies of populations overburdened with environmental toxicants.

### 3.5. Interventions

Eleven studies investigated the use of Finnish saunas and 25 studies utilised infrared sauna interventions. The remainder 4 studies used other forms of dry sauna (Thai-style or mixed). Sauna sessions varied from 5 minutes to 20 minutes in single or multiple sessions totaling 30 minutes–4 hours daily, once to several times each week over study durations that ranged from 3 days to 5 months. The cohort studies followed frequent infrared sauna bathers for 5 years and frequent male Finnish sauna bathers for over 20 years.

All of the studies involving Finnish-style saunas used interventions ranging in temperature from 80 to 90°C with relative humidity levels of 10–20% except Hüppe et al. 2009, a study comparing detoxification protocols, which employed a lower temperature sauna at 50–65°C with 30% relative humidity for 15 minutes in one intervention arm [68].

Of the 25 studies involving infrared sauna, all used far-infrared types except Ross and Sternquist 2012, which employed a full-spectrum infrared sauna as part of a detoxification protocol for policemen [69]. All infrared sauna studies entailed sauna exposures at 60°C for 15–30 minutes with the exception of two studies: Amano et al. 2015 studying the effects of sauna on patients diagnosed with chronic fatigue syndrome/myalgic encephalomyelitis (CFS/ME) using saunas set at 40°C–45°C for 15 minute sessions [54] and Oosterveld et al. 2009 examining the effects of sauna set at 55°C for 30-minute sessions on patients diagnosed with Ankylosing Spondylitis and Rheumatoid Arthritis [53].

All of the sauna interventions were conducted in supervised settings (i.e., in-hospital, rehabilitation hospitals, health centres, university or medical laboratories, and outpatient programs) except Kanji et al. 2015, which provided sauna voucher cards to allow participants to attend saunas of choice attached to local swimming pools [50] and the two large cohort studies that followed Finnish men attending saunas of their choice [38, 39].

### 3.6. Outcome Measures

Some studies focused solely on measuring subjective quality of life and symptom scoring surrounding sauna activity such as SF-36 (36-item short form health survey); FASE (Foundation for Advancements in Science and Education) 50-item survey of symptoms and sleep, CMI (Cornell Medical Index) survey of somatic complaints; VAS (visual analogue scales) for hunger, relaxation, and specific types of pain (i.e., leg pain); various numeric rating scales for pain, fatigue, sleep quality, and common cold symptoms; validated tools for depression, anxiety, headache disability, and anger such as POMS (profile of mood states) questionnaire, BDI (Beck Depression Inventory), SRQ-D (self-rating questionnaire for depression), Zung SDS (self-rating depression scale), STAI (state-trait anxiety inventory questionnaire), and HDI (Headache Disability Index) [43, 50, 51, 54, 55, 69].

Other interventional studies focused on obtaining objective measures related to sauna activity. For example, the studies involving CHF patients tracked combinations of physiological changes using body weight, body temperature, HR (heart rate) or PR (pulse rate) and SBP and DBP (systolic and diastolic blood pressures); exercise tolerance using the 6 MWD (6-minute walking distance) and peak VO_2_ (peak/maximum volume of oxygen) on bicycle ergometer; cardiomegaly/heart enlargement using CTR (cardiothoracic ratio) on CXR (chest X-ray); cardiac flow performance using standard ECHO (echocardiogram) Doppler ultrasound parameters; overall functional state using clinician-based NYHA (New York Heart Association) classification; endovascular reactivity using FMD (flow-mediated dilation of brachial artery); heart failure activity using plasma levels of BNP (B-natriuretic peptide); autonomic nervous system and immune-mediated activity using ECG (electrocardiogram) recordings with heart rate variability parameters and plasma levels of norepinephrine, TNF-*α* (tumour necrosis factor-alpha), and CD34+ (cluster of differentiation 34, bone marrow derived) cells; endovascular activity using plasma levels of VEGF (vascular endothelial growth factor), nitric oxide metabolites (nitrate and nitrite), and reactive oxygen metabolites (hydroperoxide) [31–33, 35, 36, 41, 42, 46, 48]. Studies involving patients with increased cardiovascular risk or studies of healthy patients with aims of detecting changes in cardiovascular risk with sauna activity used some of the same physiological parameters mentioned above as well as serum lipid profiles (total cholesterol, LDL, HDL, and triglycerides), fasting plasma glucose levels, serum levels of uric acid (potential marker of insulin resistance and metabolic syndrome), plasma levels of ghrelin, serum levels of leptin, plasma levels of Hb (hemoglobin) and HCT (haematocrit), and urinary prostaglandin levels [37, 47, 49, 63, 66, 67].

Other specific objective outcome measures performed before/after sauna include myocardial perfusion scintigraphy with adenosine, treadmill exercise stress test results, flow-mediated vasodilation of brachial artery, and expression of CD34-positive bone marrow-derived cells in hospital patients with ischemic heart disease and total coronary occlusion; standard spirometry parameters, peak nasal inspiratory flows, and ECG (electrocardiogram) with HRV (heart rate variability) parameters in participants diagnosed with allergic rhinitis; plasma volume changes (calculated from hemoglobin readings), hydration status using urine specific gravity, exercise performance on ergometer, and ECG with HRV parameters in elite athletes; axillary body temperatures, venous blood gas panels, lipid peroxidation by UV (ultraviolet light) and fluorescence analysis, and nitric oxide levels in elite athletes; transepidermal water loss, stratum corneum hydration, skin erythema, skin surface pH, surface sebum contents, and NaCl (sodium chloride) concentrations in sweat of healthy men and women; basic physiological observations (temperature, heart rate, blood pressure, and body weight), calculated plasma volumes, and serum levels of thyroid function (TSH (thyroid stimulating hormone), T3, and T4) and other hormones (human growth hormone, adrenocorticotropic hormone, and cortisol) in healthy women; and pre-and postintervention semen analysis including standard sperm parameters, sperm chromatin structure analysis, sperm apoptosis, quantitative sperm heat-stress gene expression levels, and plasma levels of male sex hormone levels (LH (luteinizing hormone), FSH (follicle stimulating hormone), testosterone, and inhibin) in healthy men.

Other interventional studies employed a combination of subjective and objective measures. Shinsato et al. 2010 and Tei et al. 2007 compared VAS for leg pain as well as 6 MWD (6-minute walking distance), ABI (ankle/brachial index), leg blood flows with Doppler laser imaging and angiography, gene expression levels of CD34+ blood cells and serum levels of VEGF, and nitrates and nitrites in patients hospitalised with peripheral artery disease [34, 45]. Kikuchi et al. 2014 and Umehara et al. 2008 assessed modified Borg dyspnoea scale or SGRQ (St George's Respiratory Questionnaire) in addition to basic physiological observations (temperature, BP, HR, respiratory rate, and O_2_ saturation), standard spirometry and ECHO parameters, 6 MWD or ergometer exercise tolerance, and plasma levels of BNP, HCT, and albumin in hospitalised patients with COPD [59, 60]. Oosterveld et al. 2009 utilised subjective VAS and validated tools of EPM-ROM (Escola Paulista de Medicina-range of motion), DUTCH-AIMS (Dutch arthritis impact measurement scales), BASMI (Bath Ankylosing Spondylitis functional index range of motion), and BASDAI (Bath Ankylosing Spondylitis disease activity index), as well as serum levels of ESR (erythrocyte sedimentation rate) [53]. Hüppe et al. 2009 used several self-assessed validated scoring tools: Beschwerden-Liste 24-item questionnaire of somatic symptoms, ADS-L/CES-D 20-item questionnaire of general depression, and SF-36 quality of life questionnaire. Objective tests of neuropsychological processing speed (GT-MT/ZVT scoring), concentration (attention test d2), memory power and speed (WL-N and WL-G scoring, resp.), and serum levels of three different PCB (polychlorinated biphenyl) congeners, hexachlorobenzene, DDT (dichlorodiphenyltrichloroethane), and DDE (p-dichlorodiphenylethylene) were measured before and/or after sauna interventions [68].

The two largest prospective cohort studies (*n* = 2315) tracked the incidence of dementia, Alzheimer's disease, and other cardiovascular disease-related outcomes such as sudden cardiac death, fatal coronary artery disease, fatal cardiovascular disease, and all-cause mortality over 20+ years, stratified by sauna bathing one time each week, 2-3 times each week, or 4−7 times each week [38, 39]. The one retrospective cohort study (*n* = 129) tracked episodes of cardiac death, cardiac events, and rehospitalisations due to congestive heart failure after completion of an in-hospital 5-day sauna program followed by twice weekly outpatient sauna activity over 5 years [44].

### 3.7. Health Outcomes

#### 3.7.1. Cardiovascular Disease

The findings of the 9 studies that researched sauna therapy for congestive heart failure (CHF) in adults culminated in the largest and most recent prospective multicentred randomized controlled trial involving 149 patients with advanced CHF that demonstrated small but improved 6-minute walking distances (−44.9 m ± SD 49.3 m, *p* < 0.05), reduced cardiothoracic ratios on chest X-ray (−1.58%  ±  SD 2.81%, *p* < 0.05) reflecting reduced heart sizes, and improved NYHA (New York Heart Association) classifications of disease (fewer class III and IV patients, *p* < 0.05) after 2 weeks of sauna therapy, all compared to no significant respective changes in a control group that received standard medical care [31].

A study of 12 infants with ventricular septal defects (VSDs) and related severe CHF (congestive heart failure) who underwent sauna bathing for 5 minutes daily for 4 weeks demonstrated decreased VSD (ventricular septal defect) shunt flow ratios (*p* < 0.05), which averted the need for surgical repair in 9 infants [41].

Another randomized controlled trial examined the effects of repeated sauna therapy on ventricular arrhythmias in 30 subjects with congestive heart failure and more than 200 premature ventricular contractions (PVCs) per 24 hours at baseline and reported significantly fewer PVCs (mean 848 ± 415 versus baseline mean 3097 ± 1033 per 24 hours, *p* < 0.01) after 2 weeks of repeated sauna sessions compared with no significant changes in a control group that received conventional medical therapy [36].

Two studies investigated the effects of repeated sauna sessions on patients with peripheral arterial disease. The first study was a pilot trial which reported decreased visual analogue scale (VAS) pain scores (*p* < 0.01), improved 6-minute walking distance (6 MWD) (*p* < 0.01), improved ankle/brachial index (ABI) (*p* < 0.01), and an increase in visible collateral vessels in ischemic legs with digital subtraction angiography (*p* < 0.01) observed after 10 weeks of repeated sauna therapy in twenty patients [45]. The second study was a randomized controlled trial (*n* = 21) which reported similar decreases in VAS (visual analogue scale) leg pain scores (*p* < 0.05), increases in 6 MWD (*p* < 0.01), and improved ABI (*p* < 0.01) in the sauna treatment group compared with no change in the control group that received conventional medical therapy. The investigators of this second study also demonstrated a 2-fold increase in mRNA CD34/GAPDH expression in peripheral blood mononuclear cells (*p* = 0.015) and increases in serum nitrate and nitrite levels (*p* < 0.05, *p* < 0.05) in the sauna group with no respective changes in the control group and no significant changes in serum VEGF levels in either group [34].

Another randomized controlled trial examined the effects of repeated sauna therapy on 24 ischemic heart disease subjects with chronic total occlusion of coronary arteries detected on coronary angiogram who had failed or rejected attempts at percutaneous coronary intervention or who had vessels deemed unsuitable for operative interventions. This study revealed that, after 3 weeks of daily (5 times weekly) infrared sessions, the scoring indices of defect reversibility on myocardial perfusion scans (summed stress scores and summed difference scores) improved (16 ± 7 to 9 ± 6, *p* < 0.01, and 7 ± 4 to 3 ± 2, *p* < 0.01) after sauna therapy but not in the control group that received standard medical care [40].

The two largest studies of this review which prospectively followed 2315 men in Finland over 20.7 years of frequent sauna bathing for cardiovascular disease-related outcomes used multivariate analysis and calculated hazard ratios (HR) adjusting for confounding factors such as blood pressure, resting heart rate, smoking status, Type 2 diabetes, previous myocardial infarction, LDL levels, and alcohol consumption. Their findings included a 66% risk reduction [HR 0.34 (0.16–0.71), *p* = 0.004] of dementia, a 65% risk reduction [HR 0.35 (0.14–0.90), *p* = 0.03] of Alzheimer's disease, a 63% risk reduction [HR 0.37 (0.18–0.75), *p* = 0.005] of sudden cardiac death, and a 40% risk reduction [HR 0.60 (0.46–0.80), *p* < 0.001] of all-cause mortality [38, 39].

#### 3.7.2. Rheumatological and Immune-Mediated Disease

A Dutch study of 34 patients diagnosed with either rheumatoid arthritis (RA) or ankylosing spondylitis (AS) reported decreased pain and stiffness in the RA (*p* < 0.05) and AS (*p* < 0.001) groups during 4 weeks of sauna therapy that was not sustained after the 4 weeks, with no changes in disease activity being detected in either group based upon range-of-motion scoring and serum levels of ESR (erythrocyte sedimentation rate) [53].

A Japanese single-group study of 44 patients diagnosed with fibromyalgia with or without another rheumatological disorder (i.e., systemic lupus erythematosus, systemic sclerosis, rheumatoid arthritis, Sjogren's syndrome, Behcet's disease, or aortitis syndrome) reported subjective improvements in VAS (visual analogue scale) pain scores (*p* < 0.001), reduced symptoms based upon FIQ (fibromyalgia impact questionnaire) (*p* < 0.001), improved quality of life indicators on SF-36 (short form 36-item) questionnaire (*p* < 0.01–0.05), and objective findings of fewer number of tender points (*p* < 0.01) palpated on physical exam after 12 weeks of combined far-infrared sauna and underwater exercise therapy [56].

Two studies of patients diagnosed with chronic fatigue syndrome/myalgic encephalomyelitis reported subjective improvements after repeated sauna. Soejima et al. 2015 (*n* = 10) reported decreased fatigue (*p* = 0.002) on numerical rating scales and improved scores for anxiety (*p* = 0.008), depression (*p* = 0.018), fatigue (*p* = 0.005), and performance status (*p* = 0.005) on POMS (profile of mood states) questionnaire after 4 weeks of infrared sauna sessions [55]. Amano et al. 2015 (*n* = 15) noted 77.8% of participants receiving 8 weeks of regular far-infrared sauna therapy improved in symptoms based upon SF-36 (short form 36-item), SRQ-D (brief self-rating questionnaire for depression), and STAI (state-trait anxiety inventory questionnaire) compared to 50% of participants in the control group, who chose not to undergo sauna therapy [54].

#### 3.7.3. Chronic Pain Syndromes

Two randomized controlled trials investigated the subjective effects of repeated sauna on chronic pain disorders. One New Zealand study (*n* = 37) of patients diagnosed with chronic tension headaches reported a 44% reduction in headache intensity within 6 weeks of the sauna treatment arm, with mean change in headache intensity between sauna and control group being 1.27 points (95% CI 0.48–2.07; *F* = 10.17; df = 1,117; *p* = 0.002) [50]. The other Japanese randomized controlled trial of 46 patients with chronic pain disorders detected an increased likelihood of return to work 2 years after sauna intervention (*p* < 0.05) and decreases in anger scoring (on CMI, Cornell Medical Index) in the 4-week sauna-treated group compared to control group (4.5 ± 1.1 to 2.2 ± 1.6, *p* < 0.001) who received the same courses of behavioural/rehabilitation/exercise therapy without additional sauna therapy [51].

#### 3.7.4. Depression

One randomized controlled trial that investigated the effects of 4 weeks of sauna sessions on 28 patients diagnosed with mild depression reported improved somatic complaints (*p* < 0.001), improved hunger scores (*p* < 0.0001), and improved relaxation scores (*p* < 0.0001) based upon subjective somatic complaint, depression, hunger, and relaxation scoring in the sauna group as compared to the control group that received bedrest instead of sauna therapy. In this same study, plasma ghrelin concentrations and daily caloric intakes also changed in the sauna group compared to control group (^*∗*^*t* = −2.32, *p* < 0.05, and ^*∗*^*t* = −2.65, *p* < 0.05, resp.) with ^*∗*^Student two-tailed group *t*-test [52].

#### 3.7.5. Lungs and Airways

Two studies focused on the effects of infrared sauna on patients diagnosed with COPD (chronic obstructive pulmonary disease). One controlled trial (*n* = 20) reported improved FEF_50_ (forced expiratory flow after 50% of expired forced vital capacity) in patients receiving 4 weeks of repeated sauna [+0.08 L/s (0.01–0.212 L/s)] versus a control group [−0.01 L/s (−0.075–0.04 L/s)], *p* = 0.019, that received usual medical care. No other changes in spirometry parameters or 6-minute walk test distances were detected between the two groups [59]. The second study involved a single group of male, ex-smoker COPD patients (*n* = 13) with the following findings after 4 weeks of sauna sessions: improved symptom scores (59.7 pts ± 16.9 to 55.3 pts ± 17.2 pts, *p* = 0.002); decreased pulmonary artery pressures during exercise (*p* = 0.028); increased exercise times after sauna exposures (360 s ± 107 s to 392 s ± 97 s, *p* = 0.032); and improved oxygen saturation during exercise (*p* = 0.022) [60].

The Thai randomized controlled trial (*n* = 26) that investigated the effects of a 6-week rehabilitation sauna program on patients diagnosed with symptomatic allergic rhinitis reported improved peak nasal inspiratory flow rates (119.2 L/s ± 46.4 to 161.9 L/s ± 46.7, *p* = 0.002) and improved FEV_1_ (forced expiratory volume at 1 sec) (77.5%  ± 9.8% to 95.6%  ± 5.7%, *p* = 0.002) in the sauna intervention group compared to a control group that received usual medical care. The researchers also examined HRV (heart rate variability) parameters but detected no significant difference between the sauna and control groups [57].

Another randomized controlled trial studied common cold sufferers in Germany (*n* = 157) sitting for 3 minutes fully winter-dressed in a Finnish sauna daily over 3 days breathing in piped “hot dry” sauna air versus control “cool dry” room temperature air while wearing a face mask. Only on day 2 assessment, a decrease in symptom severity scoring was detected in treatment versus control groups [−1.0 (−2.0–−0.1), *p* = 0.04, 95% CI] but this finding was not sustained through days 3, 5, and 7 of study. Fewer doses of cold and flu medications were taken by the treatment group on day 1 of assessment [3% (1–9%) versus 15% (8–28%), *p* = 0.01, 95% CI], compared to the control group [58].

#### 3.7.6. Athletes

Two small noncontrolled interventional trials studied the physiological effects of repeat sauna in athletes. One study (*n* = 7) reported that 30 minutes of daily postexercise sauna bathing for ten days was associated with peaked expansion of plasma volume after 4 days (+17.8%, 90% CI: 7.4–29.3%), followed by a trend back to presauna levels by days 7–10 [61]. The other study (*n* = 16) noted a mean postsauna increase in axillary body temp 2.6°C (*p* < 0.001) after first sauna versus a mean increase of only 1.9°C (*p* < 0.002) after completing a 5 months' course of sauna bathing. The researchers also noted postsauna increases in mean venous pH by 0.8% (*p* < 0.001), decreased mean base excess by 20.3% (*p* < 0.001), increased mean venous O_2_ by 53.3% (*p* < 0.001), increased mean Hb concentration in blood by 5.2% (*p* < 0.001), and right shift of oxygen-hemoglobin dissociation curve (decreased affinity, favours release of O_2_ to tissues) after the first sauna with similar changes in specified parameters noted after a final sauna 5 months later (*p* < 0.043–*p* < 0.005) [62].

#### 3.7.7. Healthy Populations

Two small uncontrolled, single-gender studies reported reduced total cholesterol levels (4.50 ± 0.66 mmol/L to 4.18 ± 0.41 mmol/L, *p* = 0.02) and reduced LDL (low density lipoprotein) levels (2.71 ± 0.47 mmol/L to 2.43 ± 0.35, *p* = 0.01) in healthy men (*n* = 16) after 4 weeks of regular sauna activity involving 45 min sauna sessions [66] and reduced total cholesterol levels (4.47 ± 0.85 mmol/L to 4.25 ± 0.93 mmol/L, *p* < 0.05) and reduced LDL levels (2.83 ± 0.80 mmol/L to 2.69 ± 0.83 mmol/L, *p* < 0.05) in healthy women (*n* = 9) after 2 weeks of regular sauna activity involving 30-minute sauna sessions [67]. The same research group of both studies reported earlier findings of significant increases in heart rate, systolic blood pressure, growth hormone, adrenocorticotropic hormone, and cortisol levels along with significant decreases in diastolic blood pressure and plasma volumes after single and repeated sauna sessions in 20 women after 2 weeks of either 30-min sauna sessions or 45-min sauna sessions [63, 65]. Reductions in total and LDL cholesterol levels along with increased HDL (high density lipoprotein) cholesterol levels were reported in the 45-min sauna group.

Another study of healthy men and women examined the skin physiology of regular sauna attenders (*n* = 21) compared to newcomer sauna attenders (*n* = 20) before and after sauna bathing. The investigators reported a decrease in NaCl (sodium chloride) sweat concentrations in the regular sauna group (~200 mmol/L ± ~10 mmol/L to ~170 mmol/L ± ~10 mmol/L, *p* = 0.0167) without any respective changes in the newcomer sauna group. Baseline values (presauna) of forehead sebum level were 25% lower in the regular sauna group (*p* < 0.05) compared with newcomer group but sebum levels decreased similarly in both groups after sauna. Skin surface pH was generally measured to be lower in the regular sauna group but similar scales of pH elevation were recorded for both groups during and after sauna activity [64].

#### 3.7.8. Detoxification

Populations burdened with toxicants were the subject of two studies. Both entailed multimodal therapies with sauna as a prominent but not sole intervention and both demonstrated improved self-assessed quality of life measures [68, 69]. Ross and Sternquist 2012 (*n* = 69) documented improved posttreatment SF-36 (short form 36-item health survey) scores in symptomatic policemen exposed to employment-related drugs and toxicants compared to pretreatment scores (with 2-tailed Student *t*-test paired scores and Wilcoxon matched pairs test and sign test, *p* < 0.001), across all subscales after 4−6 weeks of infrared sauna sessions with up to 4 hours of sauna bathing daily. The FASE (Foundation for Advancements in Science and Education) 50-item and neurotoxicity symptom questionnaires further revealed fewer “poor physical health” days (9.3 versus 1.8 days, *p* < 0.001); fewer “sick days” (2.0 versus 0.3 days, *p* < 0.001); more sleep hours (5.8 versus 7.6 h, *p* < 0.001); and lessened neurotoxicity scoring (65.5 ± 24.8 versus 14/6 ± 11/5 points, *p* < 0.001) [69].

The other sauna detoxification study was a randomized controlled trial (*n* = 36) of symptomatic individuals with elevated levels of lipophilic toxicants, comparing two separate sauna interventions with a control group: (I) steam sauna with oral and intravenous supplements, (II) dry sauna with substitute placebo oral and intravenous interventions, and (III) no sauna, no oral, and no intravenous interventions. Using multivariate analysis of variance (MANOVA) methods, several somatic well-being scores improved in both treatment groups (I) and (II), as compared to group (III) with Duncan post hoc test suggesting significant differences between group (I) and group (III) (*p* < 0.01) and between groups (I) and (II) (*p* < 0.05). No differences however were seen between groups (II) and (III) (*p* = 0.21) and no significant changes in neuropsychological testing scores (*p* > 0.10) or serum concentrations of selected organochlorides (*p* > 0.10) were reported between any of the groups [68].

#### 3.7.9. Spermatogenesis

One longitudinal time-course study examined the effects of Finnish sauna activity on male sperm and fertility measures in 10 healthy men. After 3 months of repeated sauna (15-min saunas twice weekly), the investigators reported reduced sperm counts (93 ± 27.0 × 10^6^ versus 223 ± 52.8 × 10^6^, *p* < 0.001); reduced sperm concentrations (31 ± 13.1 × 10^6^/ml versus 89 ± 29.3 × 10^6^/ml, *p* < 0.001); fewer motile sperm (36.1 ± 3.6% versus 58.0 ± 7.6%, *p* < 0.01); abnormal sperm parameters [decrease in normal histone-protamine replacement (*p* < 0.05); abnormal chromatin condensation (*p* < 0.05); altered mitochondrial function (*p* < 0.01)]; upregulation of various heat-stress genes [HIF-1*α* (*p* < 0.001), KDR (*p* < 0.001), FLT1 (*p* < 0.001), and VEGF (*p* < 0.001)]; and upregulation of HSPs (heat shock proteins) and HSFs (heat shock factors) [HSP90 (*p* < 0.001), HSP70 (*p* < 0.001), HSF1 (*p* < 0.001), HSF2 (*p* < 0.001), and HSFY (*p* < 0.001)]. However, all specified changes reverted back to normal 6 months after ceasing sauna activity and no significant changes in plasma sex hormones from baseline were detected directly after sauna or after 3 or 6 months [27].

#### 3.7.10. Adverse Side Effects

Of the 40 included studies, only eight reported any adverse symptoms from sauna bathing. Six studies recorded adverse effects graded as mild, meaning symptoms of complaint were noted which did not alter the study protocol or incur dropouts to the study. Mild heat discomfort was the major complaint [53, 61, 69]. Other mild complaints noted in one infrared sauna study of heart failure patients (*n* = 149) included symptomatic low blood pressure, hypovolemia, polyurination, weight loss, and, questionably, acute bleeding after a tooth extraction [31]. Another study of patients with peripheral arterial disease (*n* = 21) reported transient leg pain in one participant during a first infrared sauna session with the pain improving after completing a few sauna sessions and disappearing altogether by the end of the 6-week study [34]. Pach et al. 2010 reported coughing in 3 of 157 Finnish-style sauna participants, stimulated by the placement of a face mask in both intervention and control groups, with different temperatures of air piped through the masks of the respective groups [58].

Two studies recorded moderate adverse effects, defined as symptom complaints that led to dropout of study participants or led to changes in study protocols. One study, involving fifteen women diagnosed with chronic fatigue syndrome/myalgic encephalomyelitis, reported enough heat intolerance in “most” of the participants such that the investigators reduced the temperature of the infrared sauna intervention from 60°C to 45°C to finish conducting the study [54]. Another infrared sauna study (randomized controlled trial) of chronic pain patients (*n* = 46) reported 2 patients dropping out of the treatment arm due to acute bronchitis and claustrophobia experienced in the sauna room [51]. None of the included studies reported severe adverse effects involving the need for emergency medical services.

## 4. Discussion

### 4.1. Principal Findings

The findings of this review suggest frequent dry sauna bathing improves a variety of subjective and objective health parameters and that frequent Finnish sauna bathing is associated with improved outcomes such as reduced overall mortality and reduced incidence of cardiovascular events and dementia, at least in men [38, 39]. The most established clinical benefits of sauna bathing are associated with cardiovascular disease, yet there is also evidence to suggest that saunas, either Finnish-style or infrared, may benefit people with rheumatic diseases such as fibromyalgia, rheumatoid arthritis, and ankylosing spondylitis, as well as patients with chronic fatigue and pain syndromes, chronic obstructive pulmonary disease, and allergic rhinitis. Sauna bathing may also improve exercise performance in athletes, skin moisture barrier properties, and quality of life and is not associated with serious adverse events. There is not yet enough evidence to distinguish any particular health differences between repeat Finnish-style and repeat infrared sauna bathing.

Cardiovascular disease has clearly been a focus for sauna researchers since 2000 despite Finnish-style sauna being considered by some in the past as a contraindication for patients with CHF and other cardiovascular diseases, most likely because of perceived intolerance to the high temperatures [1]. Nearly half (19 of 40) of the studies included in this review involved populations who had active cardiovascular disease or increased risk for cardiovascular disease, and all these studies demonstrated beneficial health effects. Seven of these 19 studies were randomized controlled trials (RCTs), with only one of them meeting the Cochrane criteria for an acceptably low risk of bias. This particular multicentre RCT (*n* = 149) reported improvements in all outcome measures except B-type natriuretic peptide (BNP) levels (namely, longer 6-minute walking distance, reduced cardiothoracic ratio on chest X-ray, and improved NYHA (New York Health Association) classification) in the infrared sauna-treated congestive heart failure group compared to control over only 2 weeks of intervention [31].

While sauna bathing appears to show promise as a lifestyle intervention for cardiovascular disease, a majority of the cardiovascular disease-related sauna studies (16 of 19) were conducted by the same core Japanese research group and affiliates who employed “Waon therapy” [74], which involved far-infrared sauna bathing. These Waon therapy studies used similar outcome measures and mostly involved hospitalised patients, which might reflect some differences in health care systems and thresholds for hospitalisation. The use of primarily hospitalised patients in these studies also brings up issues of how applicable the findings may or may not be to outpatient populations.

Despite differences in sauna types, temperature, frequency, and duration of interventions, the far-infrared sauna studies involving cardiovascular disease and congestive heart failure patients suggest favourable outcomes that reinforce earlier findings of interventional Finnish sauna studies and cardiovascular disease [75–79]. This suggests that heat stress, whether induced by infrared or Finnish-style sauna, causes significant sweating that is likely to lead to hormetic adaptation and beneficial cardiovascular and metabolic effects. This is further supported by the two large observational studies that found striking risk reductions for sudden cardiac death (63%) and all-cause mortality (40%) as well as for dementia (66%) and Alzheimer's disease (65%), in men who used a sauna 4−7 times per week compared to only once per week [38, 39]. While these large cohort studies are based on calculated hazard ratios with adjustments for common cardiac risk factors, it has been pointed out that the association between sauna activity and health outcomes may be noncausal and that sauna use is merely an indicator of “healthy lifestyle” and other socioeconomic confounding factors [80]. Nevertheless, these findings point to the need for further study and serious consideration given to sauna bathing to address the ever-increasing individual, societal, and financial burdens of cardiovascular disease as well as dementia-related conditions in aging populations.

### 4.2. Mechanisms of Action: Sauna Bathing

Several mechanisms of action have been proposed for the health effects of frequent sauna bathing. Exposure to heat increases cardiac output and reduces peripheral vascular resistance and induces other physiological changes in cardiovascular parameters such as decreased systolic and/or diastolic blood pressure [31, 35, 37, 46–49, 60, 65], increased HRV (heart rate variability) [33, 36, 57], improved cardiac function markers [31, 33, 35, 36, 40, 42, 46, 48], and improved flow-mediated arterio- and vasodilatation of small and/or large blood vessels [34, 40, 42, 45, 47–49]. Regarding hormonal and metabolic models, reduced levels of epinephrine and/or norepinephrine [42, 46], increased levels of nitric oxide metabolites in blood [32, 34] and urine [41], decreased total and LDL (low density lipoprotein) cholesterol levels [63, 66, 67], increased serum levels of growth hormone, adrenocorticotropic hormone (ACTH), and cortisol [65], decreased fasting blood glucose levels [49], increased plasma ghrelin levels [52], and reduced urinary levels of prostaglandins (8-epi-prostaglandin F_2*α*_) [37] have been detected after regular sauna sessions. Together, these findings support complex multipathway end-organ effects on the central and autonomic nervous system, the peripheral vascular endothelium, and the hypothalamus-pituitary-adrenal axis, as well as on the kidneys and the liver that are continuing to be documented [1, 11, 28, 81].

The complexity of how sauna bathing may influence cardiovascular risk factors is suggested by the report of beneficial effects on total cholesterol and LDL (low density lipoprotein) cholesterol and conflicting results on HDL (high density lipoprotein) levels in healthy young men and women [63, 66, 67]. These findings, which need to be confirmed in larger studies with nonsauna control groups, may point to differences between Finnish and infrared saunas as they contrast with previous similarly sized, yet better controlled studies of infrared sauna bathing in populations at increased risk of cardiovascular disease [37, 47, 49]. These findings may also be compared to the metabolic effects of exercise in healthy populations which include improvements in both LDL and HDL lipid levels [82].

While there are likely to be many mechanisms of action influencing the physiological effects of sauna bathing, it has been suggested that sauna bathing may induce a general stress-adaptation response that leads to “hormetic adaptation” and the establishment of “sauna fitness,” possibly analogous to the hormetic adaptation responses of exercise. This is supported by newer, single-cell analysis methods that suggest sauna bathing increases generation of free radicals and reactive oxygenated species along with enhanced antioxidant activities via proposed nitric oxide- (NO-) dependent processes in blood [62] and upregulation of specific HSPs (heat shock proteins) and HSFs (heat shock factors) in semen [27]. The two studies in athletes further support sauna's involvement in hormetic stress responses with the findings of plasma volume expansion after 4 days of daily postexercise sauna bathing, followed by a trend back to presauna levels by days 7–10 in one study [61], along with mean postsauna increases in axillary body temperature of 2.6°C after a first sauna versus mean postsauna increases of only 1.9°C after the last session of a 5-month course in the other study [62]. Additionally, increases in plasma lipid peroxidase levels and increases in free radical processes of RBCs and decreases in plasma *α*-tocopherol (antioxidant) levels and decreases in RBC catalase activity after an initial sauna were not maintained after 5 months of regular sauna [62], suggesting that sauna bathing may upregulate antioxidant defences.

Improved adaptation to stress with regular sauna bathing may be further enhanced by excretion of toxicants through heavy sweating. Many industrial toxicants including heavy metals, pesticides, and various petrochemicals may be excreted in sweat leading to an enhancement of metabolic pathways and processes that these toxic agents inhibit [83]. Sweat-induced excretion of toxic metals such as arsenic, cadmium, lead, and mercury has been reported with the rates of excretion matching or exceeding urinary routes [84]. There is also recent evidence that toxic chemicals and xenobiotics such as polybrominated diphenyl ether (PBDE) flame retardants, organochlorine pesticides, bisphenol-A (BPA), and phthalates may be excreted via induced sweating at rates that exceed urinary excretion [85–88]. The importance of sweat in excretion pathways has been further documented by sweat-patch technology used to monitor illicit drug use and is based on dozens of studies of the pharmacodynamics and pharmacokinetics of amphetamine, cocaine, cannabis, opiates, and associated metabolites [89, 90]. While sweat-induced detoxification certainly occurs, studies using sauna for detoxification purposes report more favourable findings with subjective rather than objective measures [68, 69]. Further research on sauna-based detoxification is warranted as the excretory functions of skin via sweating or other active, passive inter- and/or transcellular, and transdermal pathways are complex and the role of frequent sweating to promote excretion and improve health is still poorly defined [91].

In addition to having profound physiological effects, sauna bathing is reported to have beneficial psychological effects that are reflected in the many reports of improved well-being, pain tolerance, and other self-assessed symptom-related scoring [34, 36, 43, 45, 46, 50–56, 58, 60, 68, 69]. The psychological impact of sauna bathing may be due to a combination of factors that include release of endorphins and other opioid-like peptides such as dynorphins [81, 92], forced mindfulness, psychological stress reduction, relaxation, improved sleep, time out from busy life schedules, placebo effects, and other aspects of individual psychological and social interactions that likely occur around frequent sauna activity. While it is difficult to distinguish between the different factors that produce positive psychological effects, such effects may enhance other physiological and metabolic benefits as they are likely to promote adherence to regular sauna activity.

### 4.3. Safety and Adverse Effects with Sauna

In the medical literature at large, there are reports of severe adverse effects from saunas that include dry sauna-induced burns [93] and myocardial ischemia (especially in patients with unstable coronary artery disease) [94], along with less frequent reports of syncope/falls [1], hypersensitivity pneumonitis (“sauna lung”) [95], nonexertional heatstroke [96], rhabdomyolysis [93], ocular irritations [97], “sauna stroke syndrome” [98], and death [99]. The risk of death from saunas was examined in retrospective population studies of frequent sauna users in Sweden and Finland, with the annual death rate from saunas being reported as 0.06 and 2 per 100,000 inhabitants, respectively, with half or more of all these deaths involving the use of alcohol and a common risk factor of sauna bathing alone [99, 100].

In this review, adverse signs and symptoms of both Finnish-style and infrared sauna bathing were reported as mild to moderate heat discomfort and intolerance in 4 of the studies [53, 54, 61, 69], low blood pressure/light-headedness in one study [31], transient leg pain in another study [34], airway irritation in two studies [51, 58], and claustrophobia in one study [51], with no severe adverse symptoms reported in any studies. Detailed comparative analysis of adverse effects between studies was limited by small sample sizes, heterogeneity of sauna types and study design (many without control groups), and inconsistent reporting of adverse side effects within outcome measures. The highest intensity of adverse effects (moderate levels of heat intolerance) occurred in populations afflicted with chronic fatigue syndrome, chronic pain, rheumatoid arthritis, and ankylosing spondylitis. As these conditions are all associated with inflammation and abnormal immune responses, it may be that the heat and/or increased sweating of sauna activity is modulating some of these responses [51, 53, 54]. The direct adverse effects of heat may also be responsible for the impairment of sperm counts, concentration, and motility and upregulation of heat-stress-related genes reported in the sperm of 10 healthy men after a 3-month course of Finnish-style sauna [27]. While these findings are based upon one identified study of only 10 men, the findings are consistent with some earlier research on the effects of genital heat stress on semen quality [101–104]. All the deleterious sperm effects of the sauna intervention mentioned in this study were observed to revert back to “normal” presauna levels after 6 months of avoiding sauna activity [27]. While this supports current recommendations for men seeking to optimize fertility to avoid sauna-type activities [105], further research is required to determine if similar effects on sperm occur with lower temperature infrared sauna bathing or if sauna bathing has any effect on male fertility.

### 4.4. Strengths/Limitations

To the best of our knowledge, this is the first systematic review to include both Finnish-style and infrared sauna studies. However, we did not include studies of steam sauna interventions and therefore may have overlooked some evidence of the effects of heat on health. Another limitation of this study is the inclusion of only English language, especially since sauna activity is frequent in non-English speaking countries. Furthermore, the quality of the reviewed studies was variable with many studies having small sample sizes, poorly described methodology, variable use of controls, differing types of sauna and sauna protocols, variable duration and frequency of sauna interventions, and inconsistent mention of cooling therapies or rehydration protocols along with heterogeneous outcome measures. The great heterogeneity of studies makes meaningful comparisons across studies difficult and provides insufficient evidence to recommend specific temperature, frequency, or duration of sauna bathing for any specific health outcome.

In the months since this systematic review was conducted, a number of new research findings have been published, analyzing various subsets of the same Finnish prospective cohort of over 2000 men who regularly sauna-bathed, initially aged 42–60 years, followed over 20 years as part of the KIHD (Kuopio Ischemic Heart Disease) study, as detailed in two of the studies included in Table 1: cardiovascular disease- (CVD-) related sauna studies. These newer findings cite reduced risk of acute and chronic respiratory conditions [106], reduced risk of pneumonia [107], reduced serum levels of C-reactive protein (marker of systemic inflammation) [108] with more frequent sauna bathing, and reduced risk of hypertension [109] and additional improved all-cause mortality when jointly associated with cardiorespiratory fitness [110]. These findings add further support to the conclusions of this review.

### 4.5. Future Research Perspectives

With the rise of single-cell analysis and “omics” platforms of analysis such as metabolomics and transcriptomics, especially applied to sweat, blood, urine, saliva, and other human biofluids, the ability to unravel the metabolic pathways at work during sauna or whole-body thermotherapy will certainly improve. Further study of these metabolic pathways might also help to elucidate the stress-related pathways of immune and inflammatory activity that may be involved in conditions such as chronic fatigue syndrome, chronic pain, rheumatoid arthritis, and ankylosing spondylitis.

Studies examining heart rate variability (HRV) as an outcome assessment are increasing and further results may better inform the physiological models of what is thought to be happening with repeated sauna of either Finnish or infrared types. The concepts of hormetic stress and interrelating “sauna fitness” or habituation to the physiological effects of repeated sauna activity might have implications for preventive or therapeutic targets in the future. Conducting more studies of repeated sauna in healthy but nonathletic participants may further help to elucidate the similarities and differences in metabolic pathways between repeated sauna activity and regular exercise. Further studies are also needed to distinguish between the health effects of Finnish saunas, which often involve brief periods of increased humidity and dramatic cooling interventions, compared to the lower temperature infrared saunas that typically do not have such variations.

## 5. Conclusions

Regular infrared and/or Finnish sauna bathing has the potential to provide many beneficial health effects, especially for those with cardiovascular-related and rheumatological disease, as well as athletes seeking improved exercise performance. The mechanisms for these effects may include increased bioavailability of NO (nitric oxide) to vascular endothelium, heat shock protein-mediated metabolic activation, immune and hormonal pathway alterations, enhanced excretions of toxicants through increased sweating, and other hormetic stress responses.

Currently there is insufficient evidence to recommend specific types of sauna bathing for specific clinical conditions. While regular sauna bathing appears to be well-tolerated in the clinical setting with only minor and infrequent adverse effects reported, further data on the frequency and extent of adverse effects is required. Further studies are also required to explore the mechanisms by which sauna bathing exerts physiological, psychological, and metabolic effects, as well as to better define the benefits and risks of distinct types of saunas and the optimal frequency and duration of sauna bathing for beneficial health effects.

## Figures and Tables

**Table 1:** Cardiovascular disease- (CVD-) related sauna studies.

Study Characteristics			Study sample		Intervention		Comparators		Health effects	Adverse side effects
Author & year	Level of evidence	Design	Pop/country	*N*	Sauna type	Duration	Comparator/ controls	Outcome measures	Positive/negative/negligible	None/mild/ moderate/ severe
2016 Tei et al. [31]	I	RCT- multicentre	Advanced CHF/Japan	149	FIR	2 weeks	Control group, standard medical care	6 MWD (6 min walking distance), CTR (cardio-thoracic ratio) on chest X-ray, NYHA class, plasma BNP levels	*Positive*, improved 6 MWD (*p* < 0.05), reduced CTR on CXR (*p* < 0.05), improved NYHA classification (*p* < 0.05) compared to control group	*Mild*, decreased BP, hypovolemia, polyurination, decreased body wt

2011 Fujita et al. [32]	I	RCT	CHF/Japan	40	FIR	4 weeks	Control group, standard medical care	Body weight, BP, cardio-thoracic ratio (CTR) on chest X-ray, LVEF on ECHO, fasting plasma levels of BNP, uric acid, hydro-peroxide, nitrate, nitrite	*Positive*, sauna group with reduced concentration of hydroperoxide (*p* < 0.001); reduced BNP levels (*p* < 0.001); increased nitric oxide metabolites (*p* < 0.05)	None

2011 Kuwahata et al. [33]	I	RCT	CHF/Japan	54	FIR	4 weeks	Control group, standard medical care	Body weight, BP, HR, CTR on chest X-ray, standard ECHO parameters, fasting plasma levels of catechol-amines and BNP; and HRV (heart rate variability) parameters	*Positive*, mean HR decreased (*p* < 0.05) in sauna group compared to control group. High frequency component of HRV in setting of beta blockade improved	None

2010 Shinsato et al. [34]	I	RCT	PAD/Japan	21	FIR	6 weeks	Control group, standard medical care	Leg pain (using VAS), ABI (ankle-brachial index), 6 MWD (6-min walking distance), PCR-CD34+ progenitor gene expression levels in peripheral blood mononuclear cells, serum levels of VEGF (vascular endothelial growth factor), nitrate, nitrite	*Positive*, decrease in leg pain scores (*p* < 0.05), increase in 6 MWD (*p* < 0.01), improved ABI (*p* < 0.01), 2-fold increase in mRNA CD34/GAPDH gene expression levels (*p* = 0.015), increases in serum nitrate and nitrite levels (*p* < 0.05, *p* < 0.05) in sauna group compared to control group	*Mild*, transient leg pain during sauna but resolved after a few sessions

2008 Miyata et al. [35]	I	RCT	CHF/Japan	188	FIR	2 weeks	Control group- standard medical care	BP, HR, body weight, body temp, CTR (cardio-thoracic ratio) on chest X-ray, usual ECHO parameters, fasting plasma BNP	*Positive*, BP and CTR decreased in both groups (sauna *p* < 0.01, *p* < 0.001; control *p* < 0.05, *p* < 0.05). Body wt decreased (*p* < 0.0001); LVEF on ECHO increased (*p* < 0.0001); plasma BNP decreased (*p* < 0.001) in sauna group compared with control group	None

2004 Kihara et al. [36]	I	RCT	Cardiac arrhythmias, CHF/Japan	30	FIR	2 weeks	Control group placebo intervention -supine on a bed in a temp-controlled room at 24°C for 45 min.	Self-assessed quality of life questionnaire, 24-hr ambulatory ECG recordings with HRV analysis (std deviation of mean RR intervals), CTR (cardiothoracic ratio) by chest X-ray, usual ECHO parameters, plasma concentrations of catechol-amines, ANP, BNP	*Positive*, fewer PVCs (*p* < 0.01), fewer couplets (*p* < 0.05), fewer episodes of VT (*p* < 0.01), decreased CTR (*p* < 0.05), increased HRV variability (*p* < 0.01), lowered serum levels of BNP (*p* < 0.01) in sauna treatment group compared to control group	None

2004 Masuda et al. [37]	I	RCT	Increased CVD Risk/Japan	28	FIR	2 weeks	Control group placebo intervention -supine on a bed in a temp-controlled room at 24°C for 45 min.	Body wt, HR, BP, HCT, fasting plasma lipid profile and glucose, urinary levels 8-epi-prosta-glandin F_2*α*_	*Positive*, systolic BP (*p* < 0.05) and urinary 8-epi- prostaglandin F_2*α*_ levels (*p* < 0.001) significantly lower in sauna group compared to control group	None

2016 Laukkanen et al. [38]	II	Prospective cohort study	Middle-aged males/Finland	2315	Finnish	20.7 years	Frequency and duration of sauna bathing: 1 time/wk, 2-3 time/wk, 4–7 times/wk	Incidence dementia/Alzheimer's disease and other CVD-related outcomes	*Positive*, sauna bathing 4−7 times a week associated with 66% risk reduction (hazard ratio 0.34, 95% CI) in developing dementia or Alzheimer's compared with 1 time/week	None

2015 Laukkanen et al. [39]	II	Prospective cohort study	Middle-aged males/Finland	2315	Finnish	20.7 years	Frequency and duration of sauna bathing: 1 time/wk, 2-3 time/wk, 4–7 times/wk	Incidence of sudden cardiac death, fatal coronary heart disease, fatal CVD, all-cause mortality	*Positive*, sauna bathing 4–7 sessions weekly associated with 40% reduction in all-cause mortality compared with 1 session weekly, (hazard ratio 0.60, 95% CI, 0.46–0.80, *p* < 0.001)	None

2013 Sobajima et al. [40]	II	Controlled clinical study	IHD with total coronary occlusion/Japan	24	FIR	3 weeks	Control group, standard medical care	Myocardial perfusion scintigraphy with adenosine, flow-mediated vaso-dilation of brachial artery, treadmill exercise stress testing and expression of CD34-positive bone marrow-derived cells	*Positive*, improved indices of defect reversibility on myocardial perfusion scans (*p* < 0.01); extended treadmill times (*p* < 0.01), improved flow-mediated dilation of brachial artery (*p* < 0.05) after sauna therapy compared to control group	None

2003 Sugahara et al. [41]	II	Single group clinical study	Infants- VSD and CHF/Japan	12	FIR	4 weeks	No control group	Core body temp, HR, BP, usual ECHO parameters including VSD measurements with colour Doppler, 24 h urine nitrate and nitrite levels	*Positive*, decrease in VSD shunt flow ratio (*p* < 0.05), increase in 24 h urine nitrite and urine nitrate levels (*p* < 0.05, *p* < 0.05); surgical repair not necessary for 9/12 (75%) infants	None

2012 Ohori et al. [42]	III	Single group clinical study	CHF/Japan	41	FIR	3 weeks	No control group	6 MWT (6-min walk test); standard ECHO parameters; plasma levels of BNP, norepinephrine and circulating CD34+ cells; flow-mediated dilation (FMD) of the brachial artery	*Positive*, increased LVEF (left ventricular ejection fraction), *p* = 0.023; reduced levels of norepinephrine and BNP, *p* = 0.015 and *p* = 0.035; increased 6 MWT, *p* < 0.001; improved FMD, *p* < 0.001; increased CD34+ counts, *p* = 0.025	None

2010 Beever [43]	III	Single group, sequential, longitudinal, interrupted time series	Type 2 diabetes/Canada	15	FIR	3 months	No control group	SF-36 (36-item short form health survey) and VAS (visual analogue scales)	*Positive*, improved stress (*p* = 0.042), fatigue (*p* = 0.014), general health (*p* = 0.037) on SF-36	None

2009 Kihara et al. [44]	III	Retrospective cohort study	CHF/Japan	129	FIR	5 years	Control group, standard medical care	Episodes of cardiac death, cardiac events, rehospitalisations due to CHF	*Positive*, 8/64 patients died in sauna therapy group vs 12/65 patients in control group (12.5% vs 18.5% mortality rate); Rehospitalization due to worsening CHF occurred in 20/64 (31.3%) patients in sauna group vs 44/65 (68.7%) patients in control group (*p* < 0.01); 38% reduction in cardiac event rate in sauna therapy group compared to control group	None

2007 Tei et al. [45]	III	Single group clinical study/pilot trial	PAD/Japan	20	FIR	10 weeks	No control group	Leg pain using VAS (visual analogue scale), 6 MWD (6 min walking distance), ABI (ankle/ brachial index), leg blood flow with Doppler laser imaging, digital subtraction angiography	*Positive*, pain scores decreased, 6 MWD improved, ABI improved, increase in visible collateral vessels in ischaemic legs with digital subtraction angiography observed after sauna therapy (*p* < 0.01 for all)	None

2005 Miyamoto et al. [46]	III	Single group clinical study/pilot trial	CHF/Japan	15	FIR	4 weeks	No control group	Body wt, BP, HR; Self-assessed quality of life questionnaire; 6 MWT (6 min walk time); peak VO_2_ on bicycle ergometer; CTR (cardio-thoracic ratio) on chest X-ray; usual ECHO parameters, plasma BNP, catecholamines; number of hospitalisations one-year after sauna intervention	*Positive*, decreased SBP (*p* < 0.05), improved CTR (*p* < 0.05), improved LVEF on ECHO (*p* < 0.05), increased 6 MWT (*p* < 0.05), decreased plasma norepinephrine and epinephrine levels (*p* < 0.01, *p* < 0.05) with sauna intervention. Reduced number of hospitalisations (*p* < 0.01) one-year after sauna intervention	None

2003 Biro et al. [47]	III	Clinical study with control group	Obesity, T2DM, smoking, hypercholesterolaemia, HTN/Japan	35	FIR	2 weeks	10/35 control group without any lifestyle diseases	Body wt, HR, BP, HCT; fasting serum lipid profile, glucose, uric acid levels; resting arterial diameter; flow mediated dilatation of brachial artery on Doppler USS; plasma ghrelin and serum leptin levels	*Positive*, decreased body wt (*p* < 0.05), SBP and DBP (*p* < 0.01, *p* < 0.05), FBG (*p* < 0.05); Improved flow mediated dilation of brachial artery (*p* < 0.001) in sauna group but results compared to control not presented	None

2002 Kihara et al. [48]	III	Clinical study with control group	CHF/Japan	30	FIR	2 weeks	10/30 control group, standard medical care	Self-assessed quality of life questionnaire; HR, BP; fasting plasma levels of catecholamines, ANP, BNP, thiobarbituric acid-reactive substances, TNF-alpha; CTR (cardio-thoracic ratio) on chest X-ray; usual ECHO parameters; brachial artery diameter and flow-mediated dilation using Doppler ultrasound	*Positive*, decreased SBP (*p* = 0.019), decreased CTR on CXR (*p* = 0.002), decreased LVEDD (left ventricular end-diastolic dimension) on ECHO (*p* = 0.047), decreased plasma BNP levels (*p* = 0.005), improved flow-mediated dilation of brachial artery on Doppler USS (*p* = 0.0006) in sauna group compared to control	None

2001 Imamura et al. [49]	III	Clinical study with control group	Increased CVD risk/Japan	35	FIR	2 weeks	Control group 10/35 without any CVD risk factors	Body wt, HR, BP; fasting serum levels of HCT, Lipid profile, uric acid, glucose, thiobarbituric acid-reactive substances; flow mediated dilation of brachial artery using Doppler USS; nitroglycerin-induced flow mediated dilation of brachial artery using Doppler USS	*Positive*, SBP and DBP reduced (*p* < 0.01, *p* < 0.05); body wt reduced (*p* < 0.05); fasting glucose levels decreased (*p* < 0.05); % flow mediated dilation of brachial artery improved (*p* < 0.001) in sauna group but no statistical report of comparisons with control group	None

CVD = cardiovascular disease; CHF = congestive heart failure; IHD = ischaemic heart disease; PAD = peripheral arterial disease; FIR = far-infrared sauna; VSD = ventricular septal defect; NYHA = New York Heart Association grading for CHF; temp = temperature; HR = heart rate; SBP = systolic blood pressure; DBP = diastolic blood pressure; wt = body weight; ECHO = echocardiogram; VAS = visual analogue scale; FBG = fasting blood glucose; BNP = B-natriuretic peptide; HCT = haematocrit.

**Table 2:** Sauna studies of rheumatological disease/chronic pain/depression.

Study characteristics			Study sample		Intervention		Comparators		Health effects	Adverse side effects
Author & year	Level of evidence	Design	Pop/country	*N*	Sauna type	Duration	Comparator/ controls	Outcome measures	Positive/ negative/ negligible	None/mild/ moderate/ severe
2015 Kanji et al. [50]	I	RCT	Chronic tension headache/New Zealand	37	Multiple types, sauna voucher cards	8 weeks	Control group received advice and education	NPRS (numeric pain rating scale), BDI (Beck Depression Inventory), HDI (Headache Disability Index)	*Positive*, 44% reduction in HA intensity in 6 weeks of treatment arm. Mean change in headache intensity between sauna and control group = 1.27 points (95% CI 0.48–2.07; *F* = 10.17; df = 1,117; *p* = 0.002)	None

2005 Masuda et al. [51]	I	RCT	Chronic pain/Japan	46	FIR	4 weeks	Control group received same course of behavioural counselling, CBT, rehabilitation, and exercise therapy	VAS for pain; pain behaviour assessment by researchers with 11-item questionnaire; Zung SDS (self-rating depression scale); anger scoring with CMI (Cornell Medical Index); sleep quality with simple 0–10 scoring; degree of satisfaction of treatments with simple numerical scoring; return to work 2 years after intervention	*Positive*, increased likelihood of return to work 2 years later (*p* < 0.05); decrease in anger scoring in sauna group compared to control (4.5 ± 1.1 to 2.2 ± 1.6, *p* < 0.001)	*Moderate*, 2 patients excluded -could not tolerate sauna - acute bronchitis and claustrophobia

2005 Masuda et al. [52]	I	RCT	Mild depression/ Japan	28	FIR	4 weeks	Control group received placebo, 45 min bedrest at 24°C and postrest shower in addition to the same rehab programs, physical therapy, occupational therapy	Somatic complaints with CMI (Cornell Medical Index); Zung SDS (self-rating depression scale); VAS for hunger and relaxation; plasma levels of ghrelin, glucose, catechol-amines; daily caloric intake.	*Positive*, improved somatic complaints (*p* < 0.001), improved hunger scores (*p* < 0.0001), and improved relaxation scores (*p* < 0.0001) in sauna group compared to control group. Plasma ghrelin concentrations and daily caloric intake increased in sauna group (^*∗*^*t* = −2.32, *p* < 0.05 and ^*∗*^*t* = −2.65, *p* < 0.05, respectively); ^*∗*^*t* = Student 2-tailed *t*-test	None

2009 Oosterveld et al. [53]	III	2 single-group (side-by-side) intervention pilot trials	Rheumatoid arthritis (RA) and ankylosing spondylitis (AS)/The Netherlands	34	FIR	4 weeks	No control group; two groups receiving same sauna intervention	VAS, EPM-ROM (Escola Paulista de Medicina range of motion), DUTCH-AIMS (Dutch arthritis impact measurement scales), BASMI (Bath Ankylosing Spondylitis functional index of range of motion), BASDAI (Bath Ankylosing Spondylitis disease activity index); serum ESR	*Positive*, pain and stiffness decreased in RA (*p* < 0.05) and AS (*p* < 0.001) groups during sauna sessions only.	*Mild*- 12−24% scoring uncomfortable on well-being scores during beginning of sauna

2015 Amano et al. [54]	III	Clinical study with control group, pilot trial	Females with chronic fatigue syndrome/myalgic encephalomyelitis/Japan	15	FIR	8 weeks	6/15 chose not to undergo sauna intervention	SF-36 survey; SRQ-D (brief self-rating questionnaire for depression); STAI (state-trait anxiety inventory questionnaire)	*Positive*, 7/9 in sauna group improved during sessions; 4/9 were still improved at follow-up 9−40 months afterwards; 2/9 non-responders. 3/6 controls receiving usual treatment improved at follow-up	*Moderate*- heat intolerance in most participants, protocol changed.

2015 Soejima et al. [55]	III	Single-group clinical study	Chronic fatigue syndrome (CFS)/Japan	10	FIR	4 weeks	No control group	Numerical rating scales for fatigue and POMS (profile of mood states) questionnaire	*Positive*, decreased fatigue (*p* = 0.002), improved POMS scores for anxiety (*p* = 0.008), depression (*p* = 0.018), fatigue (*p* = 0.005) and performance status (*p* = 0.005) after sauna	None

2011 Matsumoto et al. [56]	III	Single-group clinical study	Females with fibromyalgia and autoimmune disorders/Japan	44	FIR	12 weeks	Sauna only one part of intervention; combined with underwater exercise therapy; no control group	VAS-visual analogue scale; no. of tender pts on clinical exam; FIQ (fibromyalgia impact questionnaire); SF-36 quality of life questionnaire	*Positive*, reduced VAS pain scores (*p* < 0.001); fewer # of tender pts (*p* < 0.01); reduced symptoms based upon FIQ (*p* < 0.001); improved quality of life on SF-36 questionnaire (*p* < 0.01–0.05) after combined sauna + underwater exercise therapy	None

FIR = Far-infrared sauna; ESR = erythrocyte sedimentation rate; VAS = visual analogue scale; CBT = cognitive behavioural therapy.

**Table 3:** Airway conditions and repeated sauna therapy.

Study characteristics			Study sample		Intervention		Comparators		Health effects	Adverse side effects
Author & year	Level of evidence	Design	Pop/ country	*N*	Sauna type	Duration	Comparator/ control	Outcome measures	Positive/negative/ negligible	None/mild/ moderate/ severe
2013- Kunbootsri et al. [57]	I	RCT	Allergic Rhinitis/ Thailand	26	Thai/Finnish	6 weeks	Control group received education and usual medical care	HRV, peak nasal inspiratory flow and usual spirometry parameters	*Positive*, reduced high-freq component (*p* = 0.003), increased low-freq component (*p* = 0.003), increased low freq: high freq ratio (*p* = 0.003) in HRV analysis; peak nasal inspiratory flow improved (119.2 L/s ± 46.4 to 161.9 L/s ± 46.7, *p* = 0.002); FEV_1_ (forced expiratory volume at 1 sec) improved (77.5% ± 9.8% to 95.6% ± 5.7%, *p* = 0.002) in sauna group compared with control group.	None

2010- Pach et al. [58]	I	RCT – Single blinded	Coryza/ common cold symptoms/ Germany	157	Finnish	3 days	Face mask breathing hot dry air at 90°C, 20% RH in treatment group; Face mask breathing cool, dry air at 24°C, 20% RH in control group.	Symptom severity scoring (0–10) on four different days; intake of common cold medications daily during week of intervention.	*Negligible*, on day 2 only, significant decrease in symptom severity in treatment vs control group [−1.0 (−2.0–−0.1), *p* = 0.04, 95% CI] but was not sustained through day 3, 5, 7 assessments. Less cold medication taken on day 1 only [3% (1–9%) vs 15% (8–28%)] in treatment vs control group (*p* = 0.01, 95% CI).	*Mild*, cough directly stimulated by face mask in both groups (2 in treatment group; 1 in control group).

2014- Kikuchi et al. [59]	II	Controlled intervention trial	COPD/ Japan	20	FIR	4 weeks	Control group received usual medical care	Spirometry parameters; 6 MWT (6-minute walk test); modified Borg dyspnea scale; oxygen saturation; PR	*Positive*, between-group improvements in FEF_50_ (forced expiratory flow after 50% of expired forced vital capacity) in sauna group [+0.08 L/s (0.01–0.212 L/s)] vs control group [−0.01 L/s (−0.075–0.04 L/s)], *p* = 0.019.	None

2008- Umehara et al. [60]	III	Single group intervention, pilot study	Male COPD Ex-smokers/ Japan	13	FIR	4 weeks	No control group	BP, PR, body wt, body temp; usual ECHO parameters; exercise tolerance by bicycle ergometer; SGRQ (St. George's Respiratory Questionnaire) symptom scores; plasma BNP, HCT, albumin before/after treatment.	*Positive*, decreased SBP and DBP (*p* = 0.002–0.0002); improvements in RV function via increased pressure differential (*p* = 0.024); Pulmonary artery pressure during exercise decreased (*p* = 0.028); increased exercise time (360 s ± 107 s to 392 s ± 97 s, *p* = 0.032); lowest SpO_2_ during exercise increased (*p* = 0.022); symptom scores improved (59.7 pts ± 16.9 to 55.3 pts ± 17.2 pts, *p* = 0.002) after sauna.	None

COPD = chronic obstructive pulmonary disease; FIR = far-infrared sauna; PR = pulse rate; HR = heart rate; BP = blood pressure; SBP = systolic blood pressure; DBP = diastolic blood pressure; wt = weight; temp = body temperature; HRV = heart rate variability; freq = frequency; RH = relative humidity; ECHO = echocardiogram; BNP = B-natriuretic peptide; E/LFTs = electrolytes with liver function tests.

**Table 4:** Repeated sauna and athletes.

Study characteristics			Study sample		Intervention		Comparators		Health effects	Adverse side effects
Author & year	Level of evidence	Design	Pop/country	*N*	Sauna type	Duration	Comparator/ controls	Outcome measures	Positive/negative/negligible	None/mild/ moderate/ severe
2015 Stanley et al. [61]	III	Single-group, interrupted time series study	Elite Athletes– Males/ Australia	7	Finnish	10 days	No control group	Plasma volume changes (calculated from Hb readings); hydration status (using urine SG by digital refractometer); ergometer exercise performance measures; HRV	*Positive*, postexercise sauna bathing increased plasma volume after 4 days of intervention (*p* < 0.01)	*Mild* – comments of “hot and very uncomfortable, but tolerable” per thermal comfort survey conducted every 5 min during sauna sessions

2012 Zinchuk and Zhadzko [62]	III	Single-group interventional study	Male Elite Athletes/ Belarus	16	Finnish	5 months	No control group	Axillary temp; venous blood gas analysis; lipid peroxidation and free radical processes by UV and fluorescence analysis of plasma and RBCs; antioxidant estimation by *α*-tocopherol fluorescence analysis of plasma and RBC catalase activity; nitric oxide metabolism by spectrophotometric methods, plasma nitrate and nitrite levels	*Positive*, increased axillary body temp 2.6°C (*p* < 0.001) after first sauna and 1.9°C (*p* < 0.002) after course of sauna; increased pH by 0.8% (*p* < 0.001), decreased base excess by 20.3% (*p* < 0.001), increased venous O_2_ by 53.3% (*p* < 0.001), increased Hb concentration in blood by 5.2% (*p* < 0.001), right shift of oxy-Hb dissociation curve (decreased affinity – favours release of O_2_ to tissues) after 1st sauna; similar changes after final sauna (*p* < 0.043–*p* < 0.005)	None

RH = relative humidity; Hb = haemoglobin; SG = specific gravity; HRV = heart rate variability; temp = temperature; O_2_ = oxygen; ROS = reactive oxygenated species; RBCs = red blood cells or erythrocytes.

**Table 5:** Sauna studies of healthy populations.

Study characteristics			Study sample		Intervention		Comparators		Health effects	Adverse side effects
Author & year	Level of evidence	Design	Pop/country	*N*	Sauna type	Duration	Comparator/ control	Outcome measures		
2010 Pilch et al. [63]	II	Two group clinical Interventional study	Healthy females/ Poland	20	Finnish	2 weeks	Group 1 intervention- sauna × 30 min; group 2 intervention-sauna × 45 min	HR, SBP, DBP, tympanic temp, rectal temp, wt; exhaled air analysis for O_2_ uptake, CO_2_ exhalation, respiratory quotient; blood analysis for Hb, HCT, calc plasma volume changes, lipid panel, free fatty acids, total free fatty acids – all measured before/after 1st sauna and final sauna	*Positive*, reduced total cholesterol (*p* < 0.05), reduced LDL cholesterol (*p* value unclear), increased HDL cholesterol (*p* < 0.05) claimed (reported numbers do not agree) in group 2 after repeat sauna.	None

2008 Kowatzki et al. [64]	II	2-group side-by-side clinical interventional study	Healthy men and women/ Germany	41	Finnish	Minimum one month of weekly sauna use in “regular sauna group”	Two groups receive the same 2-session sauna intervention: Group 1:“regular sauna group” before intervention Group 2: “newcomer sauna group” with no prior sauna 3 months before intervention.	TEWL (trans epidermal water loss); stratum corneum hydration; skin erythema; skin surface pH; surface sebum content; ionic concentration of NaCl in sweat	*Positive*, baseline values (pre-sauna) of forehead sebum level 25% lower in regular sauna group (*p* < 0.05); sebum levels decreased similarly in both groups; decrease in NaCl sweat concentration in regular sauna group only (~200 mmol/L to ~170 mmol/L, *p* = 0.0167); skin surface pH lower in regular sauna group but similar elevations with sauna activity.	None

2007 Pilch et al. [65]	II	Two group clinical interventional study	Healthy women/ Poland	20	Finnish	2 weeks	Group 1 intervention- sauna × 30 min; group 2 intervention-sauna × 45 min	HR, BP, rectal and tympanic temp, body wt; blood Hb; calc plasma volume; serum levels of TSH, T3, T4, human growth hormone, ACTH, cortisol	*Positive*, increased HR, increased SBP, decreased DBP and reduced plasma volumes after single and repeated sauna sessions in both groups (*p* < 0.005–*p* < 0.01). Increased secretions of growth hormone, ACTH, cortisol after single and repeated sauna sessions in both groups (*p* < 0.01–*p* < 0.05).	None

2014 Gryka et al. [66]	III	Single group clinical study	Healthy males/ Poland	16	Finnish	4 weeks	No control group	Body mass, HR, Body skinfold thickness, blood lipid profiles and plasma volumes	*Positive*, reduced total cholesterol (4.50 ± 0.66 mmol/L to 4.18 ± 0.41 mmol/L, *p* = 0.02) and LDL levels (2.71 ± 0.47 mmol/L to 2.43 ± 0.35, *p* = 0.01) after 10 sessions of sauna over 2 weeks – returned to baseline after 2 weeks without sauna. No significant changes in HDL levels	None

2014 Pilch et al. [67]	III	Single group clinical study	Healthy females/ Poland	9	Finnish	2 weeks	No control group	Tympanic temp, rectal temp, wt; plasma levels of Hb, HCT, lipid panel and free fatty acids	*Positive*, reduction in total cholesterol (4.47 ± 0.85 mmol/L to 4.25 ± 0.93 mmol/L, *p* < 0.05) and LDL levels (2.83 ± 0.80 mmol/L to 2.69 ± 0.83 mmol/L, *p* < 0.05) after repeated sauna	None

HR = heart rate; SBP = systolic blood pressure; DBP = diastolic blood pressure; temp = body temperature; wt = body weight; Hb = haemoglobin; HCT = haematocrit; calc = calculated; lipid panel = total cholesterol, triglycerides/triacylglycerols, high-density lipoproteins, low-density lipoproteins; NaCl = sodium chloride. ACTH = adrenal corticotrophic hormone.

**Table 6:** Repeat sauna therapy and detoxification.

Study characteristics			Study sample		Intervention		Comparators		Health effects	Adverse side effects
Author & year	Level of evidence	Design	Pop/country	*N*	Sauna type	Duration	Comparators/ controls	Outcome measures	Positive/negative/ negligible	None/mild/ moderate/ severe
2009- Hüppe et al. [68]	I	RCT	Symptomatic patients with elevated serum levels of lipophilic toxicants (PCBs, DDT, DDE)/ Germany	36	Two types: Sauna I (65°C, 70% RH) and Sauna II (50°C, 30% RH)	4 weeks	3 groups: (I) - Steam sauna + physiotherapy + oral and intravenous detox supplements (II) - Dry sauna + physiotherapy + placebo oral and placebo intravenous supplements (III) - No sauna or oral/IV treatment	Psychologist (blinded)-assessed and self-assessed scoring using validated tools: somatic symptom complaint list scoring, Beschwerden-Liste 24-item questionnaire; general depression scoring using ADS-L/CES-D 20-item questionnaire; SF-36 quality of life questionnaire; neuropsychological processing speed with GT-MT/ZVT scoring; concentration with “attention test d2”; memory power and speed with WL-N and WL-G scoring; serum levels of PCB congeners × 3, HCB, DDT, DDE.	*Positive*, improvements in several somatic well-being scores in both treatment groups (I) & (II), as compared to group (III) with Duncan post hoc test suggesting differences between Group (I) and Group (III) (*p* < 0.01) and between Group (I) and (II) (*p* < 0.05) but no difference between Group (II) and (III) (*p* = 0.21); No significant changes in neuropsychological testing scores between the groups (*p* > 0.10); No significant changes in serum concentrations of selected organochlorides between the groups (*p* > 0.10).	None

2012- Ross and Sternquist [69]	III	Retrospective chart review and follow-up surveys	Symptomatic police officers with employment-related drug and toxicant exposures/ U.S.A.	69	Sauna with full-spectrum infrared (160°F)	4−6 weeks	No control group	RAND© SF-36 (36-item quality of health survey); FASE 50-item survey of symptoms and sleep; 13-item neurotoxicity questionnaire; MMSE; and review of daily medical records during therapy.	*Positive*, improved post treatment SF-36 scores compared to pre-treatment scores (with 2-tailed student *t*-test paired scores + Wilcoxon matched pairs test and sign test, *p* < 0.001), across all subscales; Comparing pre and post completion of program: fewer “poor physical health” days (9.3 vs 1.8 days, *p* < 0.001); fewer “sick days” (2.0 vs 0.3 days, *p* < 0.001); more sleep hours (5.8 vs 7.6 h, *p* < 0.001); lessened neurotoxicity scoring (65.5 ± 24.8 vs 14/6 ± 11/5 points, *p* < 0.001); no changes in MMSE (29.3 vs 29.1 points, *p* = 0.122).	*Mild*, heat discomfort

FASE = Foundation for Advancements in Science and Education; MMSE = Mini-Mental State Examination; ADS-L/CES-D = Allgemeine Depressions Skala/Centre for Epidemiological Studies Depression Scale; GT-MT/ZVT = German Trail-Making Test/Zahlenverbindungstest; WL-N = Wortliste Niveau memory power test; WL-G = Wortliste Geschwindigkeit memory speed test; PCB = polychlorinated biphenyls; HCB = hexachlorobenzene; DDT = Dichlorodiphenyltrichloroethane; DDE = p-dichlorodiphenylethylene.

**Table 7:** Repeated sauna and male fertility.

Study characteristics			Study sample		Intervention		Comparators		Health effects	Adverse side effects
Author & year	Level of evidence	Design	Pop/country	*N*	Sauna type	Duration	Comparator/controls	Outcome measures	Positive/negative/negligible	None/mild/ moderate/ severe
2013 Garolla et al. [27]	II	Single-group, longitudinal time-course study	Healthy males/Italy	10	Finnish sauna	3 months	No control group	Before, after intervention, after 3 months, after 6 months' intervention: semen analysis; plasma sex hormone levels (LH, FSH, testosterone, inhibin); sperm parameters; sperm chromatin structure analysis; sperm apoptosis; sperm heat stress gene expression with quantitative real-time PCR analysis: HIF-1*α*, KDR, FLT1, VEGF, HSP90, HSP70, HSF1, HSF2, HSFY	*NEGATIVE* - Post-intervention: lowered sperm count (93 ± 27.0 × 10^6^ vs 223 ± 52.8 × 10^6^, *p* < 0.001); lowered sperm concentration (31 ± 13.1 × 10^6^/ml vs 89 ± 29.3 × 10^6^/ml, *p* < 0.001); fewer motile sperm (36.1 ± 3.6% vs 58.0 ± 7.6%, *p* < 0.01) with no differences noted by 6 months post end of sauna intervention. No significant changes in plasma sex hormones at any timepoints. Abnormal sperm parameters [decrease in normal histone-protamine replacement (*p* < 0.05), abnormal chromatin condensation (*p* < 0.05), altered mitochondrial function (*p* < 0.01)]; up-regulation of heat-stress genes [HIF-1*α* (*p* < 0.001), KDR (*p* < 0.001), FLT1 (*p* < 0.001), VEGF (*p* < 0.001)] and up-regulation of heat shock proteins/factors [HSP90 (*p* < 0.001), HSP70 (*p* < 0.001), HSF1 (*p* < 0.001), HSF2 (*p* < 0.001), HSFY (*p* < 0.001)] directly after sauna intervention but all changes completely reversed by 6 months post ceasing sauna activity.	None

LH = luteinizing hormone; FSH = follicle stimulating hormone; PCR = polymerase chain reaction; HIF-1*α* = hypoxia-inducible factor I alpha; KDR = kinase insert domain; FLT1 = fms-related tyrosine kinase; VEGF = vascular endothelial growth factor; HSP90 = heat shock protein 90; HSP70 = heat shock protein 70; HSF1 = heat shock factor 1; HSF2 = heat shock factor 2; HSFY = heat shock factor Y.

**Table 8:** Risk of bias assessment in randomized controlled trials.

	Random sequence generation	Allocation concealment	Blinding of participants and personnel	Blinding of outcome assessment	Incomplete outcome data	Selective reporting	Other bias	Jadad et al. score [72]
Fujita et al. 2011	*✕*	?	*✕*	*✕*	✓	✓	?	<3
Hüppe et al. 2009	*✕*	?	✓	✓	✓	*✕*	?	<3
Kanji et al. 2015	✓	✓	✓	✓	✓	✓	✓	4
Kihara et al. 2004	*✕*	?	*✕*	*✕*	✓	?	?	<3
Kunbootsri et al. 2013	*✕*	?	*✕*	*✕*	✓	✓	?	<3
Kuwahata et al. 2011	*✕*	?	*✕*	*✕*	✓	✓	?	<3
Masuda et al. 2004	*✕*	?	*✕*	*✕*	✓	✓	*✕*	<3
Masuda et al. 2005 -pain	✓	?	*✕*	*✕*	✓	✓	*✕*	<3
Masuda et al. 2005 -depression	*✕*	?	*✕*	*✕*	✓	✓	*✕*	<3
Miyata et al. 2008	*✕*	?	*✕*	*✕*	✓	✓	?	<3
Pach et al. 2010	✓	?	✓	✓	✓	✓	?	5
Shinsato et al. 2010	*✕*	?	*✕*	*✕*	✓	✓	?	<3
Tei et al. 2016	✓	?	*✕*	✓	✓	✓	?	3

✓: low risk of bias; *✕*: high risk of bias; ?: unclear risk of bias.
